# The effect of hand position on perceived finger orientation in left- and right-handers

**DOI:** 10.1007/s00221-017-5090-5

**Published:** 2017-09-19

**Authors:** Lindsey E. Fraser, Laurence R. Harris

**Affiliations:** Department of Psychology, Center for Vision Research, York University, 4700 Keele St, Toronto, ON M3J 1P3 Canada

**Keywords:** Proprioception, Orientation, Finger, Hands, Perception

## Abstract

In the absence of visual feedback, the perceived orientation of the fingers is systematically biased. In right-handers these biases are asymmetrical between the left and right hands in the horizontal plane and may reflect common functional postures for the two hands. Here we compared finger orientation perception in right- and left-handed participants for both hands, across various hand positions in the horizontal plane. Participants rotated a white line on a screen optically superimposed over their hand to indicate the perceived position of the finger that was rotated to one of seven orientations with the hand either aligned with the body midline, aligned with the shoulder, or displaced by twice the shoulder-to-midline distance from the midline. We replicated the asymmetric pattern of biases previously reported in right-handed participants (left hand biased towards an orientation ~30° inward, right hand ~10° inward). However, no such asymmetry was found for left-handers, suggesting left-handers may use different strategies when mapping proprioception to body or space coordinates and/or have less specialization of function between the hands. Both groups’ responses rotated further outward as distance of the hand from the body midline increased, consistent with other research showing spatial orientation estimates diverge outward in the periphery. Finally, for right-handers, precision of responses was best when the hand was aligned with the shoulder compared to the other two conditions. These results highlight the unique role of hand dominance and hand position in perception of finger orientation, and provide insight into the proprioceptive position sense of the upper limbs.

## Introduction

Many tasks require us to keep track of our limbs with partial or no visual feedback. For example, a driver may operate the steering wheel of their vehicle, change gear, and activate the turn signal all while their gaze is directed at the road. A proprioceptive sense of limb position is generated by integrating peripheral signals from stretch receptors in the muscles, skin and joints, as well as central signals such as motor commands and perceived effort (Winter et al. 2005; Proske and Gandevia 2009; Smith et al. 2009; Medina et al. 2010; for a review, see Proske and Gandevia 2012). Behavioural research has found systematic biases in participant-reported hand position when visual feedback is absent. Recently, we found that errors in perceived index finger orientation were biased towards specific angles that varied as a function of plane of operation (frontoparallel or horizontal) and hand tested (left or right) in right-handed individuals (Fraser and Harris 2016). It has been suggested that perceived hand location is biased towards relevant or likely manual workspaces (Ghilardi et al. 1995; Haggard et al. 2000; Rincon-Gonzalez et al. 2011); our findings extended this theory by suggesting perceived finger orientation similarly deviates towards common functional postures of the hands (Fraser and Harris 2016). In the present study, we compared perceived finger orientation of left- and right-handed individuals in various hand locations in order to further test this possibility.

Several behavioural measures have been used to examine the accuracy and precision of proprioceptive hand and arm position sense. For example, Haggard and colleagues asked participants to mark the location of their unseen hand on the underside of a table using a pen held in the fingers of the opposite hand; perceived hand location was biased further to the left for the left hand, and further to the right for the right hand (Haggard et al. 2000). Other studies using similar reaching tasks have found similar biases, as have tasks asking participants to report static arm position with respect to a visual or proprioceptive target (Van Beers et al. 1998; Jones et al. 2010). In contrast, Schmidt and colleagues measured arm position sense using a paradigm where the participant’s forearm was slowly rotated about the elbow and the participant indicated when their arm passed under an LED. In this paradigm perceived location of the arm was biased inwards (towards the body) in right-handers (Schmidt et al. 2013). Ghilardi et al. used a reaching paradigm where participants moved their unseen hand to a visual target and also found reach biases consistent with a bias in initial hand position in towards the body midline (Ghilardi et al. 1995).

The inconsistency in the direction of reported hand location biases may be due in part to differences in hand posture and task demands. For instance, Jones et al. (2012) tested participants’ ability to reach to a target, reach to the remembered location of a reach target (reach reproduction), or judge the location of a remembered reach target with respect to a visual stimulus. They found errors similar to Haggard et al. (2000) in the reaching and location estimation tasks, but not in the reach reproduction task. These results suggest that conscious proprioceptive limb position sense may be biased while movement reproduction mechanisms remain accurate (e.g., Vindras et al. 1998).

The evidence described above suggests that there may be a systematic error in the proprioceptive mapping of the hands in space. It is known that training and experience can lead to shifts in proprioceptive localization (Cressman and Henriques 2009) and proprioceptive acuity (Wong et al. 2011). The study by Ghilardi et al. found that following training in a novel workspace, participants’ limb position estimates shifted towards this space (Ghilardi et al. 1995), indicating the location of relevant manual tasks and workspaces can influence proprioceptive maps in a dynamic way.

Recently we reported that in right-handers, the perceived orientation of the index finger of the left hand was biased towards ~25° inwards, while the right hand was biased towards only ~2° inwards, when the hand was held pronate in the horizontal plane (i.e., palm-down; Fraser and Harris 2016). These angles, which we termed the “axes of least error”, reflect hand positions commonly adopted by right-handers when doing bimanual tasks in this plane (Sainburg 2002, 2005). That is, right-handers tend to stabilize an item with their left hand (a piece of paper, a loaf of bread), and manipulate tools with their right (a pen, a bread knife). We suggested that these common, hand-specific behaviours lead to a priori assumptions about the likely orientation of the fingers of that hand, that in turn affect proprioceptively judged finger orientation estimates. Proprioceptive mapping of hand position in space does appear to be idiosyncratic, with individual participants’ localization errors remaining stable over time (Rincon-Gonzalez et al. 2011). However, our previous data (Fraser and Harris 2016) suggest that, at least for perceived finger orientation, significant group-level trends do exist. These group-level characteristics may be driven by shared experience with common everyday manual tasks, such as writing.

If perceived orientation of the fingers were biased towards common functional hand postures, we would expect to see a reversal of the errors found in right-handers (Fraser and Harris 2016) for a group with reversed functional roles of the hands, i.e., left-handers. That is, if the dominant and non-dominant hands were biased towards unique positions based on their common roles, these biases should be reversed in groups with reversed dominant and non-dominant hands. However, there is evidence that, compared to right-handers, left-handers are actually more accurate in judging the length of their arms (Linkenauger et al. 2009), represent the space around their body more evenly (Hach and Schütz-Bosbach 2010) and are more accurate in sensing their arm position (Schmidt et al. 2013). These differences may be due to reduced hemispheric lateralization in left-handers (Przybyla et al. 2012; Vingerhoets et al. 2012), and/or to greater flexibility in switching between dominant and non-dominant hands to accommodate a “right-handed world”. Some authors have argued that left-handers may use different strategies for estimating limb position, such as pictorial representation of the body (Gentilucci et al. 1998; Schmidt et al. 2013). Therefore it is possible that left-handers may not show the opposite pattern to right-handers in their perception of finger orientation. In order to address this we compared the perceived orientation of the fingers in right- and left-handed individuals with the goal of better understanding the factors contributing to proprioceptive finger orientation sense. We predicted that right-handers would show a pattern of responses similar to those reported in Fraser and Harris (2016), while left-handers would show a different, potentially reversed pattern of errors.

Additionally, some studies have reported that hand localization errors are reduced for locations closer to the body midline (Wilson et al. 2010; Rincon-Gonzalez et al. 2011). We tested right- and left- handed individuals’ perceived finger orientation when the hand was located directly in front of the body midline, aligned with the shoulder, or displaced to the ipsilateral side, to determine whether errors in reported finger orientation increased with the whole hand’s distance from the body midline for both groups. We predicted that orientation estimates would become less accurate and less precise as the distance of the hand from the body midline increased, consistent with a near-field advantage in both handedness groups.

## Methods

### Participants

Forty-one participants (14 male, 27 female) completed the study. Twenty participants scored left-handed on the Edinburgh Handedness Inventory (Oldfield 1971); the other 21 participants scored right-handed. An additional three participants scored in the middle range on the inventory (i.e., between −28 and 48; Cohen 2008) and were removed from the study. Details of the participant groups are given in Table 1.

**Table 1:** A breakdown of participant characteristics by group (left- or right-handed)

	*n*	Age	Handedness score
Left-handers	20 (5 males)	Median = 18 Range = 17–24	Mean = −86.5 Range = −50 to −100
Right-handers	21 (9 males)	Median = 20 Range = 17–40	Mean = 89.0 Range = 60–100

Handedness score based on the Edinburgh Handedness Inventory (Oldfield 1971)

All participants were graduate or undergraduate students of York University, recruited by word of mouth or through York’s Undergraduate Research Participant Pool (URPP). Students recruited through the URPP were awarded a credit towards their Introduction to Psychology grade in exchange for their participation. All other participants received no compensation. All participants reported normal or corrected-to-normal visual acuity. Written informed consent was obtained from participants prior to data collection. This study was approved by the research ethics committee at York University, and was run in accordance with the principles outlined in the Declaration of Helsinki.

### Apparatus

**Fig. 1:**
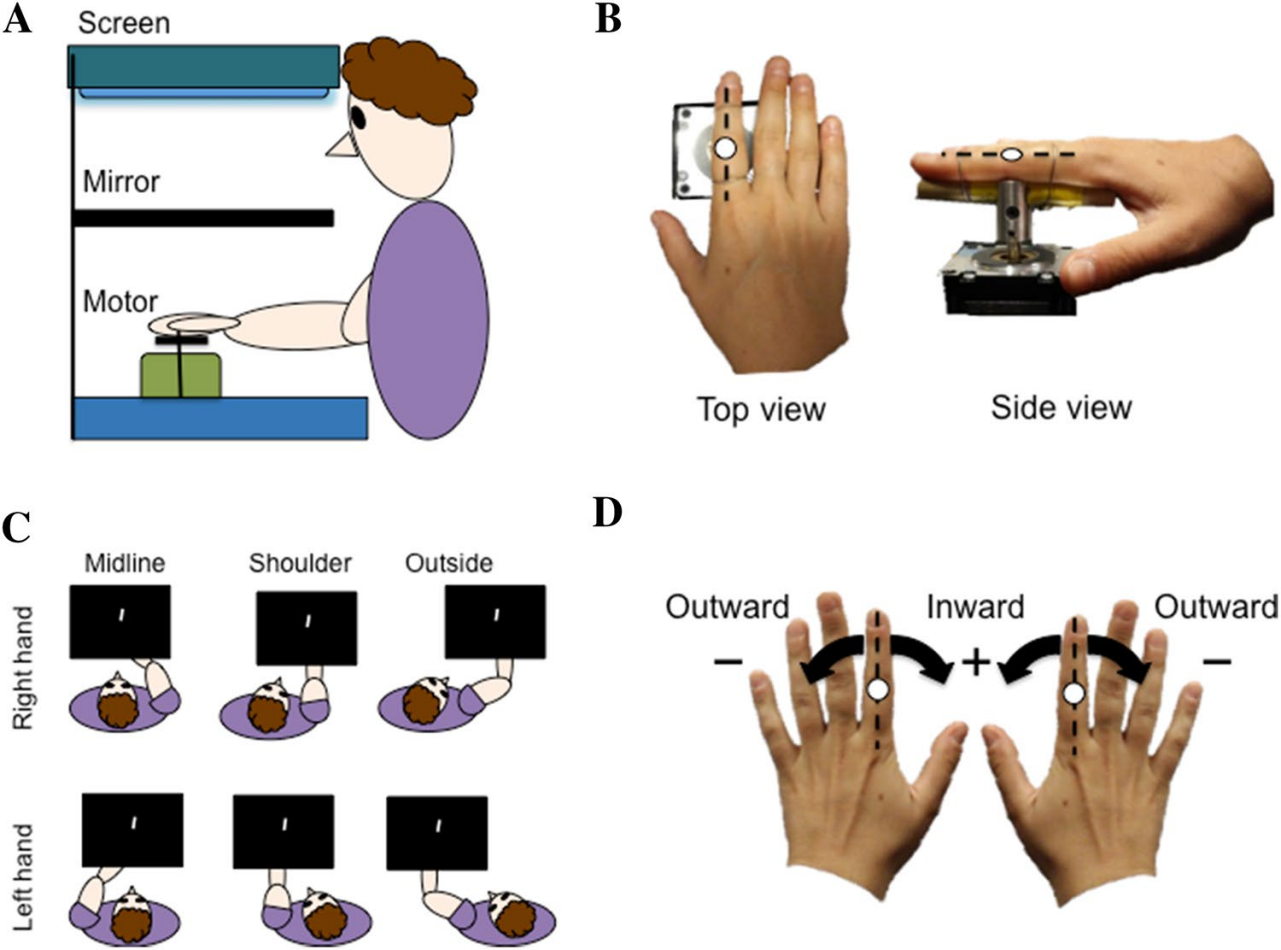
**a** A schematic of the experimental apparatus in profile. The mirror optically superimposed the screen at the location of the participant’s hand. **b** A close-up image of a participant’s left hand attached to the motor, seen from above and from the side. The axis of rotation was orthogonal to the proximal interphalangeal (PIP) joint of the index finger, denoted by a white circle in the image. **c** The six test conditions in Experiment 1 (2 hands × 3 hand locations). **d** Convention for reporting the orientation of the fingers. When the index finger is pointing straight ahead with respect to the body (as shown) it is positioned at 0°

The apparatus used in this experiment is the same as the “horizontal configuration” described in Fraser and Harris (2016). Participants were seated in front of a table on which rested a motor, a monitor and a mirror (Fig. 1a). The motor (Applied Motion Products 23Q-3AE Integrated Stepper Motor, 20,000 steps per revolution) was positioned 35 cm away from the participant with the shaft sitting 15 cm above the table surface, pointing towards the ceiling. A 5 cm wooden dowel was fastened to the motor shaft orthogonal to the axis of rotation. During the experiment the participant’s index finger was attached to this dowel with two lengths of flexible wire such that the axis of rotation passed through the proximal interphalangeal joint (PIP) of the finger, with the hand supinate (palm facing downward) (Fig. 1b). The motor was connected via a serial port to a laptop that controlled the rotation of the motor by means of a custom written program running in MATLAB. The motor was pre-set to accelerate and decelerate at 0.5 revolutions/s^2^ to a maximum velocity of 0.5 revolutions/s. The motor had a resolution of 0.02° and emitted minimal noise while in motion that gave no indication as to the direction of movement. This apparatus served to passively rotate the participant’s finger, and by extension the rest of the hand, about an axis orthogonal to the index finger’s PIP joint, to orientations specified by the test program (–30° to 30° in 10° steps, with 0° corresponding to straight ahead with respect to the body; see convention below). Pilot testing found that finger rotation to angles greater than 30° was painful and introduced large changes in elbow position to avoid discomfort, which is why test orientations were restricted to this range in this study.

A mirror was mounted horizontally half way between a horizontally mounted monitor and the shaft of the motor, obscuring the participants’ hand from view (Fig. 1a). The monitor (ASUS VS247H-P 23.6″ widescreen LCD) faced downwards such that images presented on the screen were reflected in the mirror and were seen at the depth of the participant’s hand. During the experiment participants were instructed to look at the images in the mirror.

The participants’ elbow was not fixed, allowing them to adopt natural arm and wrist positions to accommodate the motion of their finger.

### Hand locations tested

Perceived index finger orientation was tested in six experimental conditions (2 hands × 3 hand locations) presented in a blocked design. Participants sat with their right or left hand located (1) directly in front of the body (“midline”), (2) aligned with the shoulder (“shoulder”) or, (3) twice the distance between the midline and the shoulder to the ipsilateral side (“outside”) (Fig. 1c).

### Measuring perceived finger orientation

Perceived finger orientation was measured by having participants align a visual line with their unseen finger. The visual line was presented on the monitor and reflected in the mirror in which participants viewed the image (Fig. 1a, 8 cm × 1.5 cm on monitor; viewing distances approximately 15 cm, midline condition; 20 cm, shoulder condition; and 30 cm, outside condition). There were seven test finger orientations for each hand location, ranging from 30° counterclockwise of straight ahead (with respect to the body) to 30° clockwise in 10° steps. The visual line initially appeared at various orientations, randomly selected from within the range of possible test orientations. Each test orientation was repeated 8 times in a block, yielding 56 trials per block. Blocks were presented in randomized order. Each trial took 10–15 s to complete and each block took 10–12 min. Prior to each test finger orientation, the motor rotated through three “distractor” orientations randomly sampled from a normal distribution with the test orientation as the mean and a standard deviation of 10°. This was done to reduce hysteresis (e.g., the effect of always rotating to the extreme orientations from the same direction).

### Procedure

Participants first signed an informed consent form and completed an online version of the original Edinburgh Handedness Inventory (developed by Oldfield 1971; adapted for online use by Cohen 2008). The experimenter then used a meter stick to measure of the distance between the spine and the outer edge of the shoulder in order to determine the hand position for the “Outside” condition.

Participants sat with their right or left index finger attached to the wooden dowel positioned to hold the hand in one of the three hand positions (midline, shoulder or outside). The mirror reflected a dark screen. The motor then rotated through three distractor orientations, followed by the test orientation. At this point participants were prompted with a 400 Hz beep to click a mouse held in their free hand and the visual line appeared onscreen optically superimposed over the location of their finger. Participants rotated the white line clockwise or counterclockwise using the left and right mouse buttons respectively until the orientation of the line matched that of their unseen finger. Participants submitted their answer by pressing the scroll wheel on the mouse, which immediately started the next trial. Participants were asked to report when they submitted an answer in error (e.g., by accidentally pressing the scroll wheel too soon) so that these trials could be removed from the data analysis. The six test blocks were conducted in a randomized order; after the 3rd block participants were offered a short break. The entire experiment took roughly 1.5 h to complete.

### Convention

The hand used was coded as right or left. Hand position was coded with respect to the body—either in front (midline), aligned with shoulder (shoulder), or outside the shoulder (outside). Finger orientation was coded in hand-centric coordinates. “Straight ahead” with respect to the body was set as 0°, with outward deviations of the hand labeled as negative and inward deviations as positive. This means that negative values reflect a clockwise deviation from straight ahead for the right hand and a counterclockwise deviation for the left hand, and visa versa for positive values. See Fig. 1d for a visual depiction of this signing convention. This convention was used in order to compare anatomically equivalent positions of the left and right hands.

### Data analysis

Each participant yielded eight responses for each tested finger orientation, in each hand position. Scores were subjected to an initial outlier analysis, where responses ±2 standard deviations from the mean of the participant’s responses for that test orientation/hand position combination were removed from the subsequent analysis. This resulted in between 6 and 10 scores out of 336 being removed for each participant.

Angular means and standard deviation of responses were calculated using the CircStat Toolbox for MATLAB (Berens 2009). Responses for a given test orientation were averaged, and then subtracted from the value of actual test orientation, yielding a signed orientation error in which positive scores corresponded to an inwards error and negative corresponded to an outwards error. We subjected these signed orientation errors to an omnibus 2 × 2 × 3 × 7 mixed-model ANOVA comparing handedness (right- or left-handed), hand used (right or left), hand position (midline, shoulder, outside), and test angle (−30°, −20°, −10°, 0°, 10°, 20°, 30°). Planned contrasts compared overall accuracy of the left vs. right hands within each group.

Additionally, we calculated the standard deviations of participants’ responses for a given test angle per condition, yielding a measure of the precision of orientation estimates. We conducted a 2 × 2 × 3 × 7 mixed-model ANOVA on the standard deviations comparing the same factors as the previous ANOVA. Planned contrasts compared precision of orientation estimates at each of the three hand positions. Where assumptions of sphericity were violated, Greenhouse–Geisser corrected degrees of freedom and *p*-values are reported.

## Results

### Signed error

**Fig. 2:**
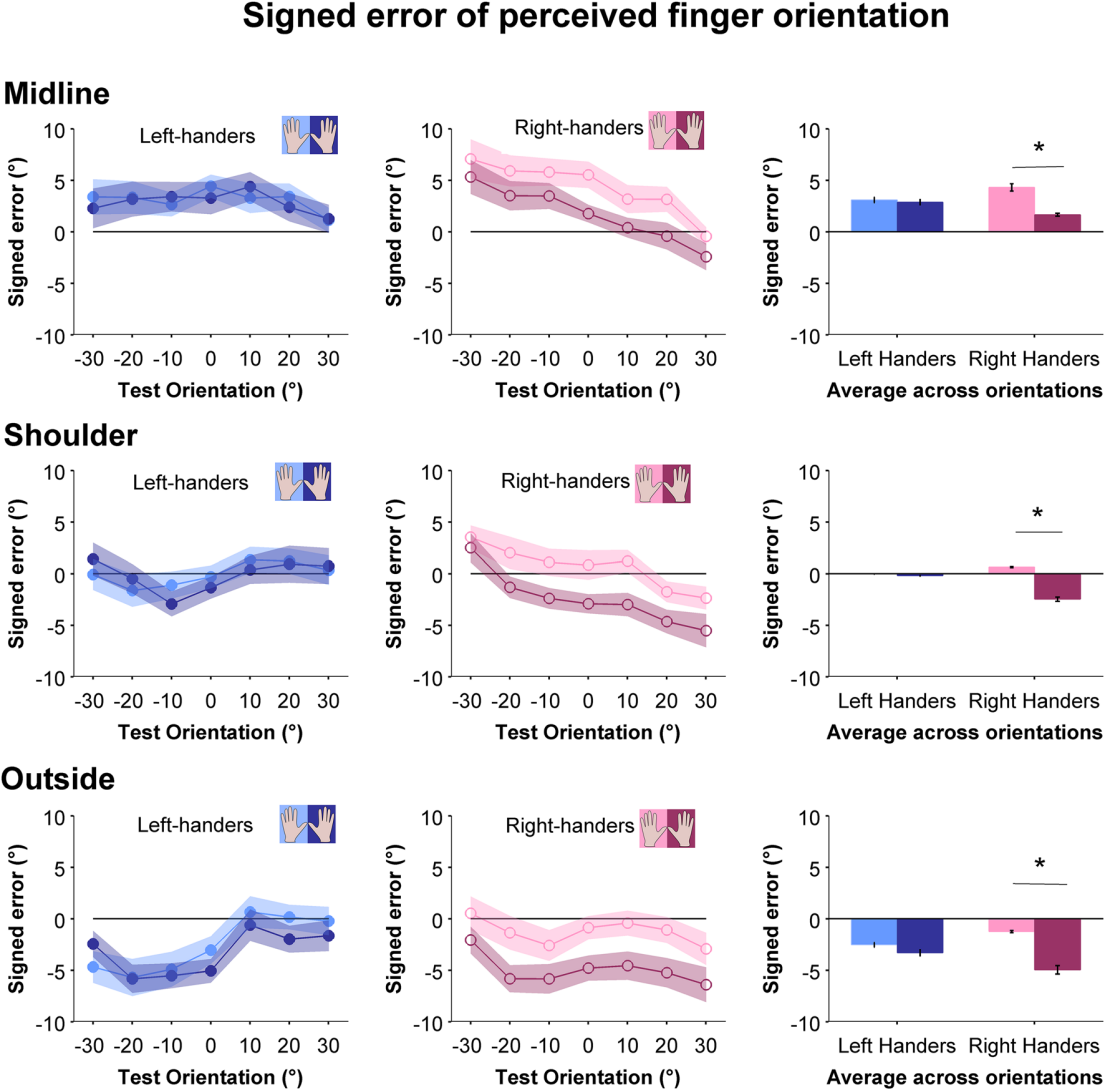
Average signed error at each test orientation for right-handers (open circles) and left-handers (filled circles) for both left and right hands The rightmost column shows signed error collapsed across tested orientations, with asterisk indicating a significant difference between hands (*p* < 0.05). For all graphs, 0° indicates straight ahead with respect to the body; negative values indicate an outward tilt of the hand (counterclockwise for left hand; clockwise for right) and positive values indicate an inward tilt (counterclockwise for right hand, clockwise for left). Therefore values lying on the horizontal line at 0° on the y axis indicate an accurate response, while values below the line indicate an outward error and those above it indicate an inward error. Shaded error zones and error bars indicate ± standard errors

Average errors for the perceived orientation of the index fingers of the left and right hands for both left and right-handers for each hand position are depicted in Fig. 2. We found a main effect of hand (left or right) *F*(1,39) = 8.60, *p* = 0.006, *η*_p_^2^ = 0.181 on mean signed error of responses. There was also a significant main effect of hand position (midline, shoulder or outside), *F*(2,78) = 40.70, *p* < 0.001, *η*_p_^2^ = 0.511. That is, signed finger orientation errors varied as a function of the hand tested and distance of the hand from the body midline. There was a significant interaction between group (right- or left-handed) and hand tested (left or right), *F*(1,39) = 5.33, *p* = 0.026, *η*_p_^2^ = 0.120, indicating responses differed as a function of which hand was tested depending on participants’ hand dominance. There was also a significant interaction between handedness and test orientation, *F*(1.53, 59.84) = 5.02, *p* = 0.016, *η*_p_^2^ = 0.114, indicating that signed error at each test finger orientation varied as a function of hand dominance. Finally, there was a significant test orientation by hand position interaction, *F*(5.88, 229.43) = 6.43, *p* < 0.001, *η*_p_^2^ = 0.142, meaning responses to specific test orientations varied as a function of hand position. No other effects or interactions were significant. Planned contrasts showed there was a significant difference between signed error for left and right hands in right-handers, *F*(1,20) = 10.41, *p* = 0.004, *η*_p_^2^ = 0.342 but not in left-handers (*p* = 0.590). Post-hoc contrasts (corrected for false discovery rate using the Benjamini–Hochberg procedure 1995) found that signed error was significantly different between the right and left hands in right-handers at each hand position, *F*(1,20) = 7.32, *p*_corrected_ = 0.018, *η*_p_^2^ = 0.268 (midline); *F*(1,20) = 6.61, *p*_corrected_ = 0.018, *η*_p_^2^ = 0.248 (shoulder); *F*(1,20) = 7.08, *p*_corrected_ = 0.018, *η*_p_^2^ = 0.261 (outside) (see Fig. 2).

### Precision of judgments

**Fig. 3:**
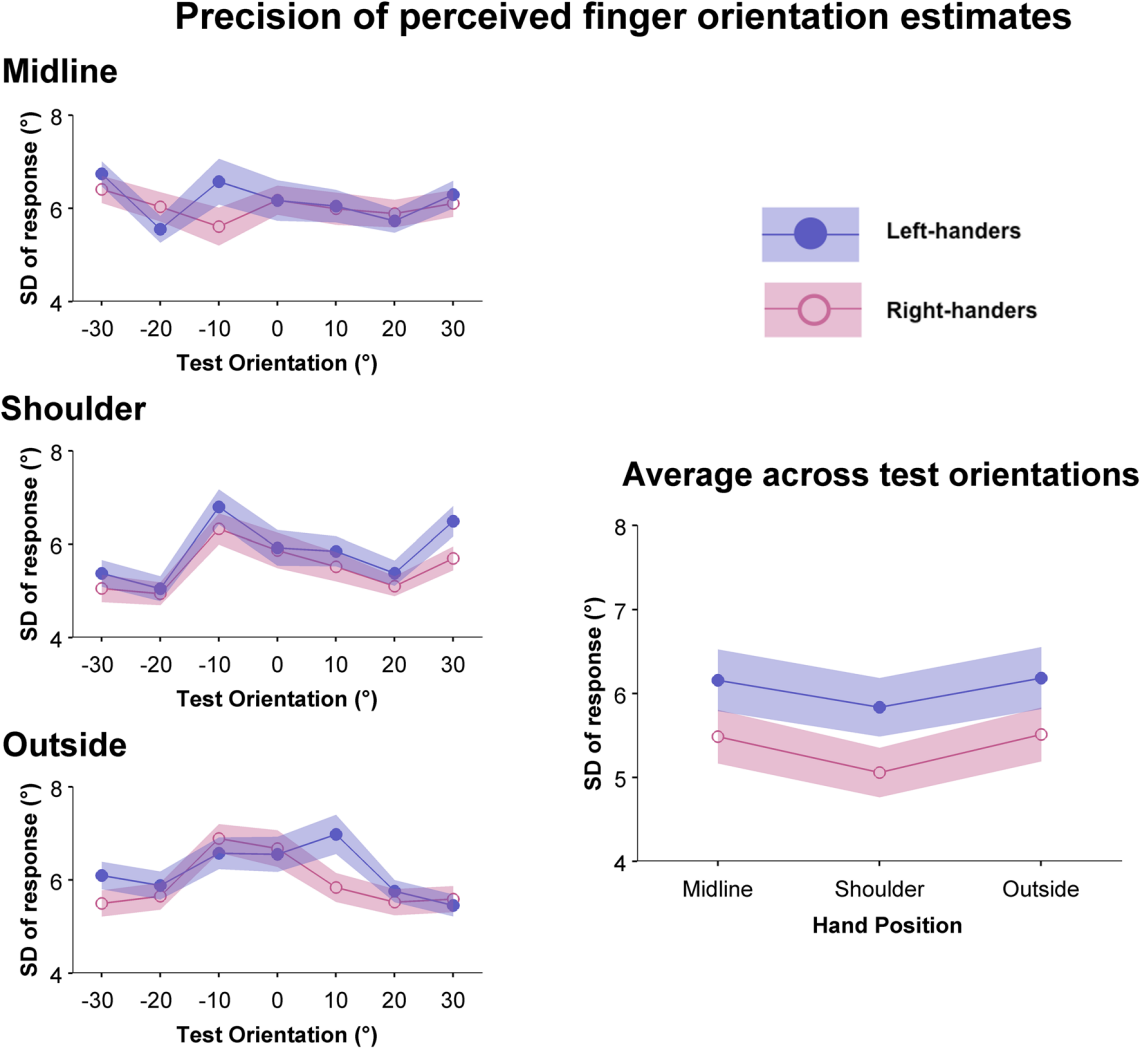
Precision of finger orientation judgments for left- (filled circles) and right-handers (open circles) at each hand position (left column), and across hand orientations (right column). Scores are collapsed across the two hands. Error zones indicate ± standard errors

The precision of finger orientation judgments is shown in Fig. 3. There was a significant main effect of position, *F*(2, 78) = 4.25, *p* = 0.018, *η*_p_^2^ = 0.098, and a significant three-way interaction between handedness, hand position and tested finger orientation, *F*(8.34, 325.33) = 1.99, *p* = 0.044, *η*_p_^2^ = 0.049. Right- and left-handers differed in precision of responses at specific test orientations and hand positions. No other effects were significant.

Planned contrasts showed that precision was best at the shoulder position (mean = 5.44 std. error = 0.22) compared to the midline condition (mean = 5.82 std. error = 0.25) *F*(1,39) = 7.63, *p* = 0.009, *η*_p_^2^ = 0.163, and to the outside condition (mean = 5.85, std. error = 0.23) *F*(1,39) = 6.65, *p* = 0.014, *η*_p_^2^ = 0.146 (see Fig. 3). Post-hoc *t*-tests (corrected with the Benjamini–Hochberg procedure for false discovery rate, Benjamini and Hochberg 1995) compared these conditions within each handedness group and found that this effect was driven by right-handers (shoulder vs. midline *F*(1,20) = 6.81, *p*_corrected_ = 0.017, *η*_p_^2^ = 0.254; shoulder vs. outside *F*(1,20) = 9.11, *p*_corrected_ = 0.017, *η*_p_^2^ = 0.313). Left-handers did not show any significant differences between these conditions.

## Discussion

We tested the perceived finger orientation of the left and right hands in left- and right-handed individuals as a function of the distance of the hand from midline. In our previous publication (Fraser and Harris 2016) we found that the perceived orientation of the index finger was biased towards specific angles for the left and right hands in right-handed individuals (~2° inwards for the right hand and ~25° inwards for the left) in the horizontal plane, when the hand was held directly in front of the body midline. We noted that these “axes of least error”, reflected commonly adopted postures of the left and right hand in right-handers (i.e., when writing or cutting). It has been established that humans have internal assumptions about the absolute and relative spatial locations of various body parts, and these assumptions can influence localization of body parts and body landmarks (Ghilardi et al. 1995; Longo et al. 2010; Longo and Haggard 2010; Medina and Coslett 2010; Longo 2015; Romano et al. 2017). Our previous study indicated that proprioceptively sensed finger orientation is similarly influenced by a priori assumptions about the likely positions of the hands (Fraser and Harris 2016). It is quite possible these assumptions are the result of repeated manual experience and learned manual behaviours, and are therefore sensitive to differences in the functional roles of the dominant and non-dominant hands. Following from this, we anticipated that left-handers might show a different pattern of errors across left and right hands compared to right-handers. Our present results reproduce the asymmetrical pattern of responses in right-handers in the horizontal plane (Fraser and Harris 2016) (see Fig. 2, middle column); however left-handers did not show any asymmetrical orientation biases between their two hands.

Further, in line with findings from other studies (e.g., Kappers and Koenderink 1999; Rincon-Gonzalez et al. 2011) we predicted that orientation estimation errors might increase as a function of the hand’s distance from the body midline. Indeed, we found that the outward rotational bias of responses increased as distance from the midline increased for both left- and right-handers, independent of the hand tested. Precision of right-handed participants’ responses was significantly better when the hand was aligned with the shoulder compared to the other two hand positions (Fig. 3); left-handers showed the same pattern in their responses, though differences between hand positions did not reach significance.

### Differences in perceived finger orientation for left- and right-handers

We found a significant interaction between hand tested and handedness in our analysis of signed errors, indicating differences between left- and right-handers’ orientation judgments of their left and right index fingers. Left-handers showed a pattern of errors that was consistent across both hands (see leftmost column in Fig. 2), with no significant differences between left and right hands and no clear axes of least error (i.e., finger orientations towards which other estimates were biased; see Fraser and Harris 2016). Therefore, left-handers did not appear to have an asymmetrical bias in the perceived orientation of their left and right fingers.

Other research has found left-handers are less systematically biased than right-handers in upper limb perception; Schmidt and colleagues tested arm position sense in left- and right-handers and found right-handers’ estimates were biased in the direction of the body midline, while left-handers’ were not (Schmidt et al. 2013). Gentilucci and colleagues have suggested that left-handers may adopt a more pictorial representation of the hands compared to right-handers, likely as a result of adapting to a right-handed world, which may lead to fewer systematic errors in perception (Gentilucci et al. 1998). Our results are compatible with a functional prior in perceived finger orientation towards specific postures only in right-handers, who may adopt a less pictorial, more function-based representation of the hands than left-handers.

Another possible explanation for our results, which is not necessarily incompatible with the theory just discussed, comes from the “dynamic-dominance theory” of arm specialization for motor control (Sainburg 2002, 2005). This theory suggests that the dominant and non-dominant limbs are specialized for different types of motor tasks; the non-dominant hand is specialized for force control and stabilization, while the dominant hand is specialized for position control. Importantly, this lateralization of function is not as pronounced in left-handers compared to right-handers, which may reflect social and environmental pressures on left-handers to operate as though they were right-handed, thus leading to more experience with motor control tasks using the non-specialized hand (Przybyla et al. 2012). Though the dynamic-dominance theory derives from the motor literature, it has implications for limb position sense in that it predicts different strategies for perceiving dominant and non-dominant hands based on what kind of sensory cues are most relevant to their specialization (vision and proprioception, respectively). For instance, Goble and Brown (2008a, b) conducted a study in which right-handed participants were asked to match their elbow angle either to (1) a remembered elbow angle presented to one of the two arms (proprioceptive cue) or (2) a visually indicated angle (visual cue). Absolute errors were smaller for the non-dominant left arm when matching a proprioceptive target, while errors for the dominant right arm were smaller when matching to the visual target. The authors suggest this difference in performance may be driven by differential weighting of cues or coordinate systems between the two hands based on their specialized roles. That is, task demands that complement the specialized role of a specific hand may lead to a handedness “advantage” in performance (Goble and Brown 2007, 2008a, b, 2010). Interestingly, the non-dominant hand “advantage” has been replicated in left-handers for joint angle matching (Goble et al. 2009) and pinch width discrimination (Han et al. 2013), i.e., tasks which require proprioceptive judgment without explicit visualization of the body. Tasks with a visual localization component, such as the one used in our study, may rely on different mechanisms (Tsay et al. 2016) that are more vulnerable to top-down influences. In other words, left-handers might still have a lateralization of function across the hands such that their non-dominant arm is still specialized for force control, but for tasks which require accessing higher-level body representations (for example visualizing a body part and comparing it to a visual target), left-handers may be less vulnerable to distortions in body image driven by functional lateralization. In line with this possibility, there is evidence that left-handers are more accurate in judging the length of the two arms (Linkenauger et al. 2009) and judging the left/right sides of their body more evenly (Hach and Schütz-Bosbach 2010) compared to right-handers.

How might a specialization of function between the two hands produce an asymmetric bias in finger orientation representation in right-handers? If we posit that different strategies or sensory weights are used to determine the orientation of the left and right fingers (for example, proprioceptive joint angle summation for stabilizing the left hand vs. coding finger endpoint location for the right), this might produce different degrees of domain-specific errors in perceived orientation. This would predict that forcing right-handers to use specific strategies for limb perception may alter the angle of least error dependent on the task in a way similar to task-dependent variation in hand-position-related errors (Jones et al. 2012).

### The effect of hand position

Figure 2 shows mean signed error collapsed across test orientations of left- and right-handers for both hands at each hand position. For both left- and right-handers, finger orientation estimates erred inwards when the hand was located on the midline. These results are inconsistent with previous research showing a “near field” advantage for accuracy of hand localization close to the body midline (Rincon-Gonzalez et al. 2011).

Figure 2 also shows that overall orientation estimates deviated inwards at the midline and rotated increasingly outwards (negative errors) as the lateral distance between the hand and the midline increased. We did not find a significant interaction between the hand’s position and any other factor in our analysis of signed errors, suggesting this rotational shift represented a constant error applied to orientation estimates for both right- and left-handed individuals, and may be the result of mechanism distinct from the source of asymmetrical hand-specific errors we saw only in right-handers.

In a series of studies, Kappers and colleagues measured haptic perception of a bar’s orientation when it was positioned in a number of different locations with respect to the body. Haptic estimates diverged outward as the distance of the bar from the body midline increased, and this result was consistent across a number of response methodologies (Kappers 1999; Kappers and Koenderink 1999; Kappers and Viergever 2006). The conclusion from these findings was that haptic space is not Euclidean in nature (Kappers 1999) and orientation errors may reflect inaccurate perception of hand orientation (Kappers and Viergever 2006). Our findings extend this work to suggest that the perceived orientation of the fingers similarly diverges as the distance between the hand and the body midline increases. Fuentes and Bastian tested proprioceptive localization of the arm about the elbow and found that more extreme joint flexion was overestimated, and this affected localization of the fingertip (Fuentes and Bastian 2010); the authors suggested this may be a safety mechanism used to protect the joints from overextension. We did not fix the elbow in our experiment (for reasons of safety and comfort), however it is a byproduct of placing the hand in front of the midline that the elbow is bent slightly inwards, and if this angle were overestimated it would also lead to an inward bias in finger orientation estimation. Similarly, when placing the hand to the side of the body the forearm is extended outwards, and if this extension were overestimated it would lead to an outward rotation of finger orientation estimates. Thus, the hand-position-related bias in finger orientation perception might be driven by more general proprioceptive mechanisms such as systematic elbow angle overestimation, which appears to affect both right- and left-handed individuals.

Finally, precision of right-handers responses was significantly better when the hand was aligned with the shoulder compared to the midline and outside conditions (Fig. 3). Left-handers showed the same pattern of changes in precision, although differences between conditions did not reach significance. The forearm’s alignment with the shoulder in this position is similar to the neutral or “orthopaedic” resting state of the arm (Bromage and Melzack 1974; Van Beers et al. 1998). There is some evidence that proprioceptive arm position sense in this range is improved compared to angles with greater muscle flexion/extension (Van Beers et al. 1998; Fuentes and Bastian 2010); this effect is not likely a direct result of differences in muscle activation between positions, as it persists when muscle load is added (Suprak et al. 2007). King and Karduna (2014) posit that neural population vectors may be more finely tuned for mid-range joint angles compared to other positions (King and Karduna 2014). Our data are consistent with this possibility, but only at the coarse level of the forearm’s position in space and not for finger orientations (compare orientations in Fig. 3). Therefore, differences in neural tuning across range of motion may only apply to gross motions like forearm abduction, and not finer movements such as precision manipulation with the fingers. Of course, targeted neurophysiological data is required to corroborate this theory.

## Conclusion

In order to manually interact with the world we must maintain a sense of our hands’ position and posture in space. Thus, a comprehensive model of proprioceptive position sense of the upper limbs must take into account the perceived orientation of the hands and fingers. This is particularly salient as proprioceptive training has been shown to improve rehabilitation outcomes following stroke and other impairments (Aman et al. 2015), and a model for healthy hand position sense, especially one that is sensitive to the roles of handedness, provides a valuable standard for both diagnosis and assessment of recovery. Our findings suggest that in the absence of vision, this perception is biased as a function of (1) handedness (and hand used) and (2) the position of the hand with respect to the body midline. That is, right-handers may adopt hand perception strategies that err towards common functional positions of the two hands, while in contrast left-handers may use a more pictorial or less functionally specific representation of their hands. Both handedness groups appear to overestimate the degree of finger rotation at positions far from the shoulder. These results highlight the uniquely lateralized nature of upper limb perception in right-handed vs. left-handed individuals; at the same time they underscore commonalities in haptic space perception between the two groups. Future work on proprioceptive and haptic space perception should carefully consider the potential contributions of the tested hand posture and hand position to final percepts.

## References

[CR1] Aman JE, Elangovan N, Yeh I-L, Konczak J (2015). The effectiveness of proprioceptive training for improving motor function: a systematic review. Front Hum Neurosci.

[CR2] Benjamini Y, Hochberg Y (1995). Controlling the false discovery rate: a practical and powerful approach to multiple testing. J R Stat Soc Ser B.

[CR3] Berens P (2009). CircStat: a MATLAB toolbox for circular statistics. J Stat Softw.

[CR4] Bromage P, Melzack R (1974). Phantom limbs and the body schema. Can Anaesth Soc J.

[CR5] Cohen MS (2008) Handedness Questionnaire. http://www.brainmapping.org/shared/Edinburgh.php. Accessed 20 June 2002

[CR6] Cressman EK, Henriques DYP (2009). Sensory recalibration of hand position following visuomotor adaptation. J Neurophysiol.

[CR7] Fraser LE, Harris LR (2016). Perceived finger orientation is biased towards functional task spaces. Exp Brain Res.

[CR8] Fuentes CT, Bastian AJ (2010). Where is your arm? Variations in proprioception across space and tasks. J Neurophysiol.

[CR9] Gentilucci M, Daprati E, Gangitano M (1998). Right-handers and left-handers have different representations of their own hand. Cogn Brain Res.

[CR10] Ghilardi MF, Gordon J, Ghez C (1995). Learning a visuomotor transformation in a local area of work space produces directional biases in other areas. J Neurophysiol.

[CR11] Goble DJ, Brown SH (2007). Task-dependent asymmetries in the utilization of proprioceptive feedback for goal-directed movement. Exp Brain Res.

[CR12] Goble DJ, Brown SH (2008). The biological and behavioral basis of upper limb asymmetries in sensorimotor performance. Neurosci Biobehav Rev.

[CR13] Goble DJ, Brown SH (2008). Upper limb asymmetries in the matching of proprioceptive versus visual targets. J Neurophysiol.

[CR14] Goble DJ, Brown SH (2010). Upper limb asymmetries in the perception of proprioceptively determined dynamic position sense. J Exp Psychol Hum Percept Perform.

[CR15] Goble DJ, Noble BC, Brown SH (2009). Proprioceptive target matching asymmetries in left-handed individuals. Exp Brain Res.

[CR16] Hach S, Schütz-Bosbach S (2010). Sinistrals’ upper hand: evidence for handedness differences in the representation of body space. Brain Cogn.

[CR17] Haggard P, Newman C, Blundell J, Andrew H (2000). The perceived position of the hand in space. Percept Psychophys.

[CR18] Han J, Waddington G, Adams R, Anson J (2013). Bimanual proprioceptive performance differs for right- and left-handed individuals. Neurosci Lett.

[CR19] Jones SAH, Cressman EK, Henriques DYP (2010). Proprioceptive localization of the left and right hands. Exp Brain Res.

[CR20] Jones SAH, Fiehler K, Henriques DYP (2012). A task-dependent effect of memory and hand-target on proprioceptive localization. Neuropsychologia.

[CR21] Kappers AML (1999). Large systematic deviations in the haptic perception of parallelity. Perception.

[CR22] Kappers AML, Koenderink JJ (1999). Haptic perception of spatial relations. Perception.

[CR23] Kappers AML, Viergever RF (2006). Hand orientation is insufficiently compensated for in haptic spatial perception. Exp Brain Res.

[CR24] King J, Karduna A (2014). Joint position sense during a reaching task improves at targets located closer to the head but is unaffected by instruction. Exp Brain Res.

[CR25] Linkenauger SA, Witt JK, Bakdash JZ (2009). Asymmetrical body perception: a possible role for neural body representations. Psychol Sci.

[CR26] Longo MR (2015). Posture modulates implicit hand maps. Conscious Cogn.

[CR27] Longo MR, Haggard P (2010). An implicit body representation underlying human position sense. Proc Natl Acad Sci USA.

[CR28] Longo MR, Azañón E, Haggard P (2010). More than skin deep: body representation beyond primary somatosensory cortex. Neuropsychologia.

[CR29] Medina J, Coslett HB (2010). From maps to form to space: touch and the body schema. Neuropsychologia.

[CR30] Medina J, Jax SA, Brown MJ, Coslett HB (2010). Contributions of efference copy to limb localization: evidence from deafferentation. Brain Res.

[CR31] Oldfield RC (1971). The assessment and analysis of handedness: the Edinburgh inventory. Neuropsychologia.

[CR32] Proske U, Gandevia SC (2009). The kinaesthetic senses. J Physiol.

[CR33] Proske U, Gandevia SC (2012). The proprioceptive senses: their roles in signaling body shape, body position and movement, and muscle force. Physiol Rev.

[CR34] Przybyla A, Good DC, Sainburg RL (2012). Dynamic dominance varies with handedness: reduced interlimb asymmetries in left-handers. Exp Brain Res.

[CR35] Rincon-Gonzalez L, Buneo CA, Tillery SI (2011). The proprioceptive map of the arm is systematic and stable, but idiosyncratic. PLoS One.

[CR36] Romano D, Marini F, Maravita A (2017). Standard body–space relationships: fingers hold spatial information. Cognition.

[CR37] Sainburg RL (2002). Evidence for a dynamic-dominance hypothesis of handedness. Exp Brain Res.

[CR38] Sainburg RL (2005). Handedness: differential specializations for control of trajectory and position. Exerc Sport Sci Rev.

[CR39] Schmidt L, Artinger F, Stumpf O, Kerkhoff G (2013). Differential effects of galvanic vestibular stimulation on arm position sense in right- vs. left-handers. Neuropsychologia.

[CR40] Smith JL, Crawford M, Proske U (2009). Signals of motor command bias joint position sense in the presence of feedback from proprioceptors. J Appl Physiol.

[CR41] Suprak DN, Osternig LR, van Donkelaar P, Karduna AR (2007). Shoulder joint position sense improves with external load. J Mot Behav.

[CR42] Tsay A, Allen TJ, Proske U (2016). Position sense at the human elbow joint measured by arm matching or pointing. Exp Brain Res.

[CR43] Van Beers RJ, Sittig AC, van der Gon JJD, Denier Van Der Gon JJ (1998). The precision of proprioceptive position sense. Exp Brain Res.

[CR44] Vindras P, Desmurget M, Prablanc C, Viviani P (1998). Pointing errors reflect biases in the perception of the initial hand position. J Neurophysiol.

[CR45] Vingerhoets G, Acke F, Alderweireldt AS (2012). Cerebral lateralization of praxis in right- and left-handedness: same pattern, different strength. Hum Brain Mapp.

[CR46] Wilson ET, Wong J, Gribble PL (2010). Mapping proprioception across a 2D horizontal workspace. PLoS One.

[CR47] Winter JA, Allen TJ, Proske U (2005). Muscle spindle signals combine with the sense of effort to indicate limb position. J Physiol.

[CR48] Wong JD, Wilson ET, Gribble PL (2011). Spatially selective enhancement of proprioceptive acuity following motor learning. J Neurophysiol.

